# Robustness of electrocardiogram signal quality indices

**DOI:** 10.1098/rsif.2022.0012

**Published:** 2022-04-13

**Authors:** Saifur Rahman, Chandan Karmakar, Iynkaran Natgunanathan, John Yearwood, Marimuthu Palaniswami

**Affiliations:** ^1^ School of Information Technology, Deakin University, Geelong 3225, Australia; ^2^ Department of Electrical and Electronic Engineering, University of Melbourne, Melbourne, Victoria 3010, Australia

**Keywords:** electrocardiogram, signal quality indices, SQA, statistical signal quality indices, threshold‌, cardiovascular diseases

## Abstract

Electrocardiogram (ECG) signal quality indices (SQIs) are essential for improving diagnostic accuracy and reliability of ECG analysis systems. In various practical applications, the ECG signals are corrupted by different types of noise. These corrupted ECG signals often provide insufficient and incorrect information regarding a patient’s health. To solve this problem, signal quality measurements should be made before an ECG signal is used for decision-making. This paper investigates the robustness of existing popular statistical signal quality indices (SSQIs): relative power of QRS complex (SQI_*p*_), skewness (SQI_skew_), signal-to-noise ratio (SQI_snr_), higher order statistics SQI (SQI_hos_) and peakedness of kurtosis (SQI_kur_). We analysed the robustness of these SSQIs against different window sizes across diverse datasets. Results showed that the performance of SSQIs considerably fluctuates against varying datasets, whereas the impact of varying window sizes was minimal. This fluctuation occurred due to the use of a static threshold value for classifying noise-free ECG signals from the raw ECG signals. Another drawback of these SSQIs is the bias towards noise-free ECG signals, that limits their usefulness in clinical settings. In summary, the fixed threshold-based SSQIs cannot be used as a robust noise detection system. In order to solve this fixed threshold problem, other techniques can be developed using adaptive thresholds and machine-learning mechanisms.

## 1. Introduction

In recent years, wearable sensors have been gaining more attention in healthcare-related applications due to their convenience of usage in daily living conditions, high availability and low cost. The demand for wearable devices currently has worldwide revenue of about $22 billion and is projected to reach approximately $45 billion ($15 billion in healthcare sector) by 2022 [1,2]. This statistic reveals the increasing demand for wearable devices, especially in healthcare. Wearable devices are available to capture different physiological signals such as electrocardiogram (ECG), photoplethysmogram (PPG) and electroencephalogram (EEG), in daily living environments.

Nowadays, a significantly larger amount of physiological signals are used in medical diagnosis [3]. The physiological signals reflect the condition of human health. Various diseases can be detected and classified by analysing these physiological signals. Although noise can interfere with different types of psychological signals such as ECG, EEG, mechanomyogram and electrooculography, in this paper, we focus on ECG signals.

ECG signals are the most frequently studied physiological signals, as they provide information about multiple physiological systems including cardiac, cardio-vascular and cardio-respiratory systems [4]. Numerous wearable ECG acquiring devices are commercially available and adopted by many researchers for clinical trials.

However, these wearable devices are very sensitive to noise due to the lower intensity of the signal. For example, noise from sensor circuits that is known as power line interference (PLI). Other noises such as baseline wander (BW) and electrode motion artefact are caused by body motion and poor electrode attachment. Some of the noise sources are uncontrollable—there include body motion, eyelid movement and device circuit noise. Therefore, it is essential to quantify the signal quality with respect to added noise before feeding the signal into a clinical decision-making system [5]. Besides noise issues, the ECG monitoring devices’ missing data are also a concern during ECG signal collection. Imputation methods are used to replace missing data points with approximate values using an autoencoder in ECG signal proposed in [6]. The imputation method did not remove noises, and it only replaced missing data points. Noise detection can be used after the imputation method. As a result, our study did not consider missing data points in ECG signals.

A noise corrupted ECG signal can cause severe consequences in a healthcare environment. For example, we assume a scenario in which a patient’s heart activities are monitored continuously and there is an automated system to alert the healthcare professionals when an heart abnormality is detected. However, this could be a false alert if the signal is corrupted by noise. Therefore, signal quality assessment is necessary before creating an alert. Furthermore, nowadays, wearable devices are connected to the Internet for remote examination by healthcare professionals. Hence, it is important to send noise-free ECG signals to those healthcare professionals. Signal quality measurements are used to determine whether an ECG signal is noisy or noise free.

### 1.1. Basic characteristics of electrocardiogram signal

**Figure 1.**
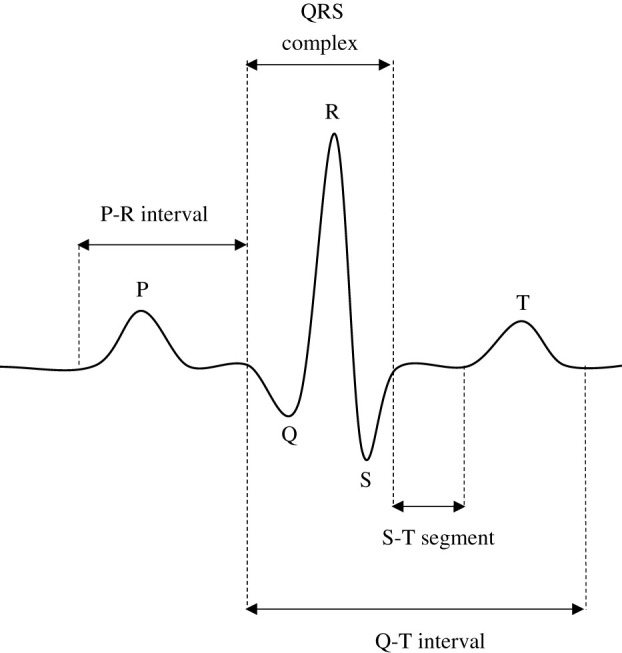
The morphological characteristics of ECG signal with S-T segment, QRS complex and Q-T, P-R interval. These intervals and segments are key to the diagnosis of heart diseases.

The heart contracts and relaxes rhythmically to pump blood around the body [7]. The sinoatrial (SA) node automatically generates electrical signals that reflect the rhythmic motion of the heart. ECG is a representation of the electrical activity of the heart, and it is used to diagnose various heart diseases. This electrical activity is recorded using electrodes placed in various places of the patient’s body [8]. An ECG signal contains feature points called P, Q, R, S and T, each representing the steps of the heart cycle as shown in figure 1. Moreover, an ECG also has five segments that are important for diagnosing different types of cardiac diseases.

### 1.2. Attributes of electrocardiogram noise

There are several types of noise in an ECG signal as described earlier. This subsection describes the noise characteristics (such as frequency range and noise source) in detail.

#### 1.2.1. Power line interference

**Figure 2.**
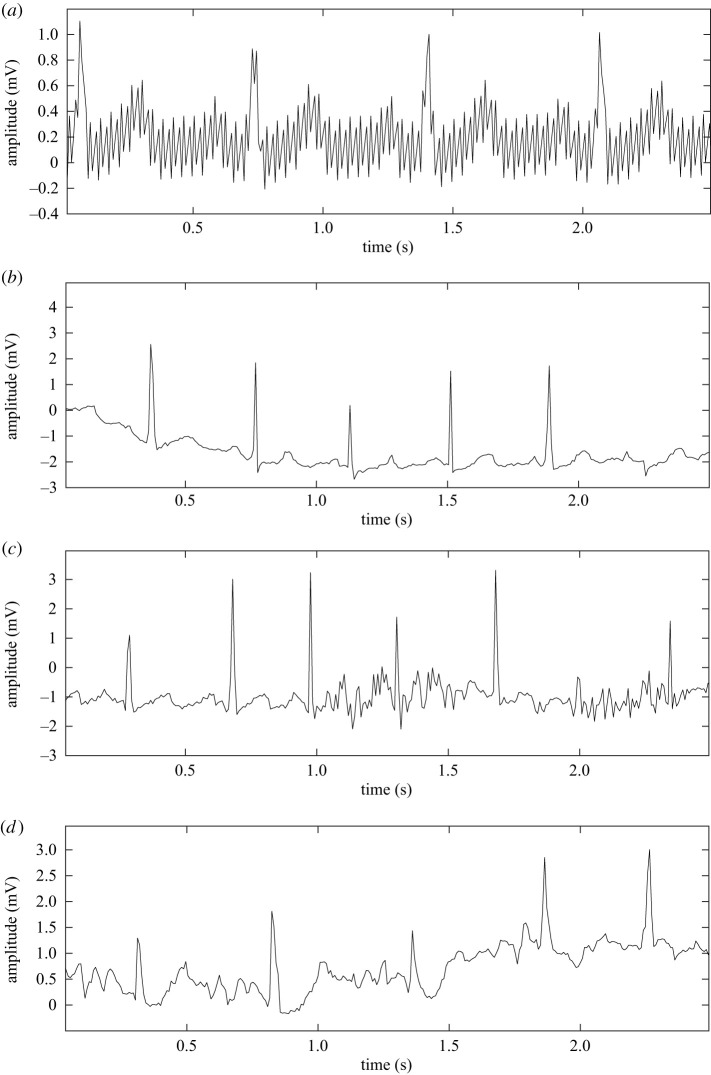
(*a*) ECG signal with power line interference, (*b*) ECG signal with baseline wander (BW), (*c*) ECG signal with muscle artefact (MA) and (*d*) ECG signal with electrode motion (EM).

One of the most common types of noise in ECG signal is power line intererence (PLI). Electromagnetic fields, power lines, poor grounding of an ECG recorder or a patient and cables loops all lead to PLI noise. It depends on the frequency (50 or 60 Hz) of the main power supply that varies from country to country. PLI distorts the P, Q, S and T peak that are important for pathological decision-making as shown in figure 2*a*.

**Figure 3.**
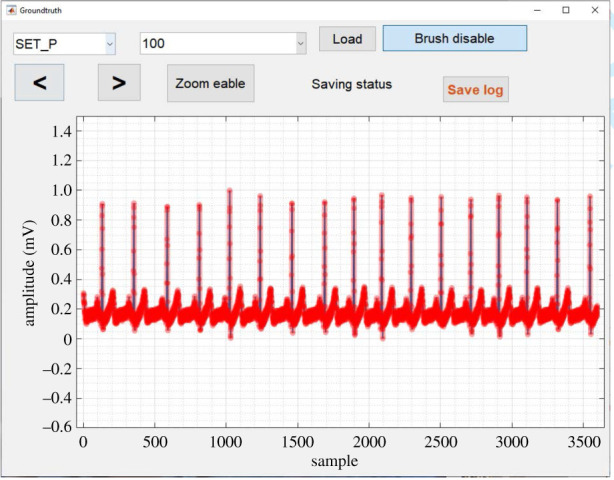
The labelling of the dataset using MATLAB GUI: red colour indicating the unacceptable and blue colour line represents the acceptable segment of the raw ECG signal.

#### 1.2.2. Baseline wander noise

Baseline wander (BW) noise is a low-frequency noise (0.1–0.2 Hz) and it is generated as a result of body movement, improper electrodes attachment, electrode–skin impedance and patient’s breathing (respiration). The shape of BW noise is a long sinusoidal signal. Because of this and the fluctuations in the ECG signal, BW noise disturbs threshold-based decision-making systems, this is illustrated in figure 2*b*.

#### 1.2.3. Muscle artefact noise

Muscle artefact noise comes from muscle movement and is depicted in figure 2*c*. Muscle movement can be divided into two parts: controllable movements (such as resting and exercising) and uncontrollable movements (such as shivering, rigours, chest compression).

#### 1.2.4. Electrode motion

Poor attachment or electrode placement in a body generates electrode motion noises and distorts the P, Q, R, S and T peaks which are presented in figure 2*d*.

#### 1.2.5. Sweating artefact

Sweating can also act as a contributor to ECG noise, especially while the ECG is used as a wearable device in daily living conditions. Sweating causes the fall of the electrode and increases skin–electrode interface impedance [9], which affect the ECG signal pattern.

### 1.3. Research gaps in electrocardiogram noise measurement

Traditionally, signal quality is improved by denoizing the signal using various filters in multiple domains such as time-domain filters (e.g. mean-median filter). In [10,11], the authors applied median and mean filters to remove BW and PLI noise. Frequency-domain filters (e.g. wavelet, low-, high-pass filter), time-frequency-domain filters (e.g. discrete cosine transform) and data-driven filters (such as empirical mode decomposition, single value decomposition) have also been used for removing ECG noise [12–15]. The major drawback of these methods is the application of the filtering technique on the entire signal without quantifying the status (noisy or noiseless) of the signal [16–20]. It is well known that filtering techniques modify phase and/or amplitude of the signal [15,21,22] and therefore, application of filtering techniques without considering the types of noise, results in distorted signals in many applications. Applying such filters on ECG signals may result in lowering the performance of decision-making models that are used for the detection or classification of different types of physiological and pathological conditions.

To address these issues, researchers have proposed various signal quality indices (SQIs) to detect the presence/absence of noise in an ECG signal rather than blindly applying filtering techniques [23–28]. This type of signal quality measurement provides necessary knowledge for the selection of an appropriate filter and the signal segment on which to perform the filtering operation. There have been several studies on SQIs of ECG in the past few years, which can be largely divided into three categories: (1) statistical SQI (SSQIs), (2) template-based SQI (TSQI) and (3) machine-learning-based SQI (MLSQI). Details of these are explained in following sectons.

Although there are many SSQI methods and their performances are reported in the existing literature, there has been little discussion about their dependency on the dataset, measurement parameter (window sizes) and rationale behind threshold selection, which determines the quality of a signal segment. Another issue that is responsible for decreasing the accuracy of signal quality prediction is accurate labelling methods. In the majority of the studies, ground truth is done by detecting QRS complexes [29–32]. The presence of a QRS complex increases the accuracy of noise and noise-free ECG signal detection. In the case of clinical usability, a QRS complex is inadequate for diagnosis of cardiac abnormalities such as first-degree atrioventricular block and left bundle branch block. As a result, the visibility of P, Q, R, S and T feature points are essential for detecting the aforementioned cardiac diseases.

A few researchers have sought to determine the standard threshold and window sizes for SSQI measurements in diverse datasets; however, they have not found a gold standard that works for multiple datasets [32,33]. In [32], the authors experimented with a fixed window size for signal quality evaluation and obtained 94.27% precision. However, in [32], different window sizes and their impact on the accuracy rate of detecting noise and noise-free signals were not considered. Nichol *et al.* [33] attempted to estimate a new threshold for the kurtosis parameter in 2018 and obtained 97% precision to distinguish a noise-free and noisy portion of the signal from each other. The major drawback of this paper is that limited datasets are used to validate their proposed threshold.

Dataset diversity is one of the major concerns for validating existing SSQI methods and its threshold values. Most researchers are focusing on ground truth labelling for part of the signal, rather than considering the entire signal. Furthermore, existing works are on limited datasets [29,32,34–36]. As a result, the effect of dataset diversity on SSQI is difficult to determine.

This indicates that further research is required for analysing the suitability of SSQI methods across different datasets.

### 1.4. Aim and contribution

The aim of this technical review is to explore the current knowledge of signal quality measurement using SSQI, analyse the robustness of existing SSQIs and highlight their strengths and weaknesses. This study examines the impact of dataset diversity and variation of window sizes on statistical signal quality determination. We have used existing SSQIs with specific window sizes (1 s, 2 s, 5 s and 10 s) where authors claimed high accuracy in [23,26,29,30,33]. For a fair comparison, we have not introduced any different window sizes other than the one that was already reported in the literature.

To accomplish that, we developed a semi-automatic tool to annotate ECG datasets based on the basic characteristics of the ECG signal. Using the annotated datasets, we analysed the performance of SSQIs across different datasets with varying window sizes. In addition, we briefly discussed other signal quality measurement techniques such as template-based SQI and highlighted their drawbacks. The major contributions of this paper can be summarized as

— Unlike the R-peak-based annotation used in the existing studies, in this study, datasets are annotated by jointly considering multiple characteristics of an ECG signal such as presence/absence of P, Q, R, S and T peaks. To facilitate annotation, we developed a user-friendly software application using MATLAB.
— This study analyses the performance of existing SQIs using six publicly available datasets. To the best of our knowledge, no other study has used so many different datasets for validation.
— This study analyses the impact of window size on the performance of SQIs. A large window size is likely unable to capture a small portion of noise. By contrast, a small window size cannot capture noise such as baseline wandering. No previous studies have explored this effect to the best of our knowledge.

The remainder of the paper is organized as follows. Section 2 discusses the signal quality measurements and their drawbacks. Section 3 presents the performance comparison of current SSQIs. The simulation results and related discussions are shown in §§4 and 5, respectively. Future directions are unlined in §§6 and 7 concludes the paper.

## 2. Electrocardiogram signal quality measurement

Signal quality assessment is a criterion for the detection of noise in an ECG signal. In an ECG signal, noise is unpredictable, and it occurs randomly. Due to this, noise can misguide QRS complex detection or signal classification. Signal quality measurement ensures the identification of noise-free ECG signals before they are used for pathological decision-making.

### 2.1. Statistical signal quality indices

SSQIs analysis is one of the pioneering and key methods to identify noisy and noise-free ECG signals [24–26,28]. The most commonly used SSQIs are relative power of QRS complex (SQI_*p*_), skewness (SQI_skew_), signal-to-noise ratio (SQI_snr_) and peakedness of kurtosis (SQI_kur_) [29,32]. Usually, higher values of SQI_*p*_ (0.5 > SQI_*p*_ < 0.8), SQI_skew_ (−0.8 > SQI_skew_ ≤ 0.8), SNR (SQI_snr_ > 10 dB) and SQI_kur_ (SQI_kur_ > 5) indicate a noise-free ECG signal.

The aforementioned popular SSQIs are briefly discussed as follows:

— **Kurtosis (SQI_kur_):** Selvaraj *et al.* observed that SQI_kur_ is an indicator of ECG signal quality [37]. Kurtosis is a statistical measure describing the distribution of the inspected data throughout the mean. It expresses a large tail and peakedness or a tiny tail and flatness of distribution corresponding to the normal distribution. SQI_kur_ can be calculated using
  2.1 $${\mathrm{SQI}}_{\mathrm{kur}}=\frac{1}{N}\sum_{i=1}^{N}{[\frac{\left(x_{i}-\bar{x}\right)}{\sigma }]}^{4},$$
  where *x* denotes the ECG signal with *N* sample points, $\bar{x}$ and *σ* represent the mean value and standard deviation of signal *x*, respectively.
— **Signal-to-noise ratio (SQI_snr_):** The SQI_snr_ is defined as the ratio of signal diversity to noise diversity [38,39]. The signal diversity represents the variance of the absolute value of the ECG signal, while the noise variance is defined as the variance of the ECG signal. In [39], the authors proposed an ECG signal with an SNR ≥ 80 dB is good quality or acceptable for further processing. The SQI_snr_ is estimated as follows:
  2.2 $${\mathrm{SQI}}_{\mathrm{snr}}=\frac{\sigma_{y}^{2}}{\sigma_{\left|y\right|}^{2}},$$
  where *y* is the ECG signal.
— **Higher-order statistics-SQI (SQI_hos_):** Nardelli *et al.* [35] proposed a novel index, SQI_hos_, using a combination of SQI_skew_ and SQI_kur_ of an ECG signal that is defined by
  2.3 $${\mathrm{SQI}}_{\mathrm{hos}}=|{\mathrm{SQI}}_{\mathrm{skew}}|\times \frac{{\mathrm{SQI}}_{\mathrm{kur}}}{5},$$
  where SQI_skew_ is denoted by
  2.4 $${\mathrm{SQI}}_{\mathrm{skew}}=\frac{1}{N}\sum_{i=1}^{N}{[\frac{\left(x_{i}-\bar{x}\right)}{\sigma }]}^{3}.$$
— **Relative power of QRS complex (SQI_*p*_):** SQI_*p*_ is the ratio between the power spectral densities of the ECG signal spectrum and the QRS complex spectrum. ECG signals are generated between 0.05 and 125 Hz for clinical analysis where the QRS complex corresponds to the frequency range [0.05 − 45] Hz. Noise-free ECG signals typically have a distinctive QRS set [23,30]. SQI_*p*_ is defined by
  2.5 $${\mathrm{SQI}}_{p}=\frac{\int_{5\;\mathrm{Hz}}^{15\;\mathrm{Hz}}P(f)\;df}{\int_{0\;\mathrm{Hz}}^{45\;\mathrm{Hz}}P(f)\;df},$$
  where *P*(*f*) is the ECG power spectrum. The majority of the ECG’s power is concentrated in the 5–15 Hz frequency range.

### 2.2. Template-based signal quality indices

Template matching is a well-known pattern recognition mechanism that is applied between a predefined signal template and a measurement signal to quantify the similarity. In [36], the authors used an adaptive template based on the QRS complex and each QRS complex is segmented as one template. In the TSQI technique, there are several distance measurement criteria, such as dynamic time wrapping (DTW), edit distance on real sequence (EDR), longest common subsequence (LCSS) and edit distance with real penalty (ERP), which are used for finding similarity between predefined templates (noise-free/noisy template) and measurement of the ECG signal [40–45]. Different distance measurement criteria are briefly discussed as follows:

— DTW matches each point to measure the distance between the predefined template and measurement signal. As a result, DTW is more sensitive to noise and needs a specific template.
— EDR, LCSS and ERP matches one point to many points between the predefined template (noise-free/noisy template) that can tolerate a small amount of noise (e.g. noise-free ECG but tolerable fluctuation).

The major concern about TSQI methods is that they work well for a given patient. However, a template developed for a patient, does not work for different patients. Therefore, it is real challenge to develop a generalized template which can work across all patients.

### 2.3. Machine-learning-based signal quality indices

In recent years, several machine-learning-based algorithms have been proposed to improve signal quality measurement [23,29]. In general, the MLSQI process depends on two major steps that are feature extraction (e.g. ECG signal features such as SQI_*p*_ and SNR) and selection of classifiers to predict signal quality from measurement of ECG signal. The most commonly used MLSQI classifiers are support vector machines (SVM), linear discriminant analysis (LDA), the multilayer perceptron (MLP) neural network, naive Bayes (NB) and convolutional neural networks (CNN) [23,27,46,47].

In [48], the authors compared four classifiers: LDA, naive Bayes, an SVM and MLP (multi-layer perception) for testing the CINC2011 dataset. In [48], firstly, 72 features (12 leads × six features) are extracted to train different algorithms for machine learning to label the data as acceptable (1) or inappropriate (−1). In the evaluation of SVM and MLP models, the authors achieved 99% accuracy when they used the CINC-2011-training (Set-a) dataset and 95% accuracy when the trained model is evaluated using the CINC-2011-testing (Set-b) dataset.

Kido *et al.* proposed a multi-class classification model [46] (qua_model) based on a CNN. The qua model for 4 s length signals will recognize the C1 class signal at a 99.00% accuracy and a 99.00% recall in 10-fold cross-validation. In [47], the authors consider the problem of five-classification classes (low interference, mild motion artefacts, mild myoelectric noise, extreme motion artefacts and extreme myoelectric noise), and a new cascaded fully CNN was proposed. Firstly, they distinguish motion and myoelectrical, and then classify the noise intensity level. The overall specificity, sensitivity and accuracy are 97.50%, 85.60% and 91.80%, respectively. The approach in [49] examines the classification of 5 s PPG segments into noise-free or noisy segments. The existing machine-learning methods on signal quality indices are presented in table 1.

MLSQI is a good technique for detecting noise in ECG segments. However, machine-learning/AI models demand more labelled datasets that are not publicly available.

## 3. Performance comparison of current statistical signal quality indices

There are several approaches for signal quality assessment, as described in §2. Previous studies indicated that SSQIs are preferable due to low complexity. However, analysis of these indices’ performance is essential for detecting noisy signals more accurately. In this section, performance and the limitations of SSQIs are analysed.

Dataset labelling is one of the critical steps for validating SQI measurements. The understanding of ECG characteristics is essential for labelling ECG signal segments as noise-free or noisy. In this section, we summarize the ECG signal features and label the ground truth by considering the morphology of the ECG signal. Finally, the SSQI parameters are described for quantifying signal quality performance.

### 3.1. Dataset labelling

In order to compare the existing SSQI, ECG datasets are labelled based on noise content and it is popularly known as an annotation. The ECG signals were manually annotated. We developed a graphical user interface (GUI) to visualize the ECG signals shown in figure 3. All the ECG segments in this study are labelled as either noise-free or noisy. The definition of noisy and noise-free ECG is defined in [52,53], where authors mentioned noisy ECG when clean ECG contaminated with PLI, BW, MA noises. Once an ECG record is selected, the GUI plots part of an ECG signal corresponding to 10 s, to visualize the feature points mentioned in §1.1. A binary array equal to the length of the uploaded ECG signal is created with binary labels, ‘1’ and ‘0’. Based on the ECG noise definition mentioned in [52,53], we assign the binary labels 'noise-free' (1) and 'noisy' (0) to parts of the ECG signal.

The buttons in the GUI can assist navigation through the ECG signal. All ECG records are labelled following similar steps using the developed GUI. Using this GUI, all the ECG signals in the dataset are binary labelled.

### 3.2. Datasets

There are several open-source ECG databases available on the Internet. The Physionet database is one of the largest ECG data providers in biomedical signal processing. We have used six datasets from the Physionet data bank. The number of subjects and total recording length of all datasets are summarized in table 2. A brief description of these datasets are presented as follows:

— **ECG-ID dataset:** the ECG-ID dataset in [55] contains 310 ECG signals, recorded from 90 patients. Each recording contains filtered and non-filtered data with 500 Hz sampling frequency. All the patients are aged between 13 and 75. The recorded channel resolution is 12-bits.
— **Tele ECG dataset:** the Tele ECG dataset in [56] contains 250 ECG signals, recorded from 120 patients using the TeleMedCare Health Monitor (TeleMedCare Pty, Ltd, Sydney, Australia). Using dry metal Ag/AgCl plate electrodes, this ECG is sampled at a rate of 500 Hz.
— **BIDMC Dataset:** the raw ECG signals obtained from clinical care at the Beth Israel Deaconess Medical Centre (Boston, MA, USA) [57]. This database includes 53 patients of different genders and ages. The duration of each record is 8 min. The sampling frequency of these signals is 125 Hz.
— **MIT/BIH arrhythmia dataset:** this collection in [55] consists of 48 patients from Boston’s Beth Israel Hospital’s Arrhythmia Laboratory. The raw signal is sampled at 360 Hz.
— **Physionet/CINC 2011 dataset:** the Physionet/CINC DB [55] comprises 1500 recordings. Each recording consists of 12 leads with 10 s recording length. The signals’ sampling frequency is 500 Hz and each sample is quantized with 16-bits.
— **Physionet/CINC 2014 dataset:** the Physionet/CINC DB 2014 comprises of 100 recordings. Each recording consists of multi-parameter records of 10 min duration and is sampled at 250 Hz.

**Table 1.** Summary of study on machine-learning-based noise detection.

method	year	model	performance			
			specificity (%)	sensitivity (%)	accuracy (%)	dataset
Tobon & Falk [50]	2015	support vector machine (SVM) and LDA	90.00-LDA	100-LDA	95.00-LDA	MITBIHA, synthetic, CINC 2011 and private
			92.00-SVM	100-SVM	96.00-SVM	
Li *et al*. [51]	2014	support vector machine (SVM)	n.a.	n.a.	80.38	MIT-BIH arrhythmia database (MITDB), CINC 2011
Behar *et al*. [29]	2013	support vector machine (SVM)	94.80	86.30	94.60	CINC 2011, MIMIC II
Clifford *et al*. [23]	2012	support vector machine (SVM)	100	99.80	99.80	CINC 2011
Li & Clifford [44]	2012	relevance vector machine (RVM)	n.a.	n.a.	86.40%	PICC, MIMIC II, Real
Kužílek *et al*. [27]	2011	support vector machine (SVM)	n.a.	n.a.	83.60	CINC 2011
Kido *et al*. [46]	2019	CNN	n.a.	97.00	n.a.	private database
Zhang *et al*. [47]	2019	cascaded CNN	97.50	85.60	91.80	MIT-BIH arrhythmia database, private dataset

n.a.: not available.

**Table 2.** Experimental datasets with a total number of subjects and each ECG signal recording length. The number of diverse datasets considered in this study compared to existing studied datasets.

ref.	database	no. subject	recording length (h)
ref. [54]	CINC 2014	200	33.33
	Telehealth ECG database	250	2.08
	total	450	35.41
[32]	artificial dataset	250	125.00
	private dataset	3	2.50
	total	253	127.50
[36]	CINC 2011	1500 (12 leads)	50.00
	MIT/BIH arrhythmia	48 (2 leads)	24.07
	MIMIC II	4050	11.25
	total	5598	85.32
[36]	CINC 2011	1500 (12 leads)	50.00
	JRD-ECG (private)	18	186.00
	total	1518	236
proposed	ECG-ID	90	0.50
	Tele ECG	250	1.96
	MIT/BIH arrhythmia	48 (2 leads)	24.07
	BIDMC	53	60.00
	CINC 2014	100	16.67
	CINC 2011	1500 (12 leads)	50.00
	total	1791	153.2

### 3.3. Segmentation

In this study, we have used four different window sizes without overlapping (1, 2, 5 and 10 s) to segment the ECG signals to analyse the influence of measurement window size.

Segmentation and labelling of segments are done automatically using the ECG record and corresponding label array. For any *n* seconds window size, the ECG record and label array are split into *n* seconds long non-overlapping segments. After segmentation, a segment is labelled as noise-free if all values of the corresponding label array segment equal one. Otherwise, the ECG segment is labelled as noisy. The number of noise-free and noisy segments for all window sizes and datasets are summarized in table 3.

**Table 3.** The comparable number of true noise-free and noisy epoch for four separate window lengths across the datasets.

epoch length (s)	dataset	no. noise-free epoch	no. noisy epoch	total epoch
	ECG-ID	1207 (67.1%)	593 (32.9%)	1800 (100%)
	Tele ECG	4357 (61.50%)	2727 (38.50%)	7084 (100%)
	BIDMC	23 361 (95.43%)	1119 (4.57%)	24 480 (100%)
1	MIT/BIH arrhythmia	144 406 (94.43%)	8518 (5.57%)	152 924 (100%)
	CINC 2011	10 528 (72.16%)	4062 (27.84%)	14 590 (100%)
	CINC 2014	47 542 (80.81%)	11 293 (19.19%)	58 835 (100%)
	ECG-ID	594 (66%)	306 (34%)	900 (100%)
	Tele ECG	2063 (59.16%)	1424 (40.84%)	3487 (100%)
	BIDMC	11 627 (94.99%)	613 (5.02%)	12 240 (100%)
2	MIT/BIH arrhythmia	72 088 (94.29%)	4363 (5.71%)	76 451 (100%)
	CINC 2011	5175 (70.94%)	2120 (29.06%)	7295 (100%)
	CINC 2014	23 623 (80.31%)	5793 (19.69%)	29 416 (100%)
	ECG-ID	230 (63.89%)	130 (36.11%)	360 (100%)
	Tele ECG	703 (52.07%)	647 (47.93%)	1350 (100%)
	BIDMC	4594 (93.83%)	302 (6.17%)	4896 (100%)
5	MIT/BIH arrhythmia	28 680 (93.83%)	1885 (6.17%)	30 565 (100%)
	CINC 2011	1987 (68.1%)	931 (31.9%)	2918 (100%)
	CINC 2014	9261 (78.72%)	2504 (21.28%)	11 765 (100%)
	ECG-ID	105 (58.33%)	75 (41.67%)	180 (100%)
	Tele ECG	233 (38.26%)	376 (61.74%)	609 (100%)
	BIDMC	2253 (92.03%)	195 (7.97%)	2448 (100%)
10	MIT/BIH arrhythmia	14 221 (93.13%)	1049 (6.87%)	15 270 (100%)
	CINC 2011	925 (63.4%)	534 (36.6%)	1459 (100%)
	CINC 2014	4507 (76.64%)	1374 (23.36%)	5881 (100%)

**Table 4.** The maximum accuracy of SQI_kur_, SQI_*p*_, SQI_snr_ and SQI_hos_ for dataset ECG-ID, Tele ECG, BIDMC, MIT/BIH arrythmia, CINC 2011 and CINC 2014.

SQI	window size/Acc.	dataset					
		ECG-ID	Tele ECG	BIDMC	MIT/BIH arrhythmia	CINC 2011	CINC 2014
SQI_kur_	window size (Sec.)	1	1	10	2	2	5
	Acc. (%)	77.96	57.48	83.95	89.58	72.33	81.23
SQI_*p*_	window size (Sec.)	2	10	10	10	2	5
	Acc. (%)	66.25	59.58	60.85	52.91	62.55	70.14
SQI_snr_	window size (Sec.)	1	5	5	2	1	1
	Acc. (%)	66.73	73.40	84.43	97.51	57.01	80.89
SQI_hos_	window size (Sec.)	2	1	2	2	1	10
	Acc. (%)	74.81	56.81	81.00	84.64	77.2	76.38

### 3.4. Performance evaluation metrics

All these traditional performance metrics depend on a confusion matrix. The confusion matrix is a way of comparing two methods of assigning a binary attribute, one of which is usually the ground-truth-based labelling and the other comes from the labelling done by SSQI indices. To evaluate model accuracy, the following parameters are used that are defined as follows:

— True positives (TP): the number of noise-free segments in true labelling estimated as noise-free segments based on the SSQIs values.
— False positives (FP): the number of noisy segments in true labelling estimated as noise-free segments based on SSQIs values.
— True negative (TN): the number of noisy segments in true labelling estimated as noisy segments based on the SSQIs values.
— False negatives (FN): the number of noise-free segments in true labelling estimated as noisy segments based on the SSQIs values.

The rate of sensitivity (Se) defines the successful separation of noise-free segments using SSQIs and measured by (3.1).

3.1 $$\mathrm{Se}=\frac{\mathrm{TP}}{\mathrm{TP}+\mathrm{FN}}\times 100\text{\%}.$$

The specificity (*S*_*p*_) is the rate of correctly detected noisy segments using SSQIs and it can be calculated by (3.2).

3.2 $$S_{p}=\frac{\mathrm{TN}}{\mathrm{TN}+\mathrm{FP}}\times 100\text{\%}.$$

Accuracy (Acc) is the relationship to the true value of the measured results and it can be calculated by (3.3).

3.3 $$\mathrm{Acc}=\frac{\mathrm{TP}+\mathrm{TN}}{\mathrm{TN}+\mathrm{TP}+\mathrm{FP}+\mathrm{FN}}\times 100\text{\%}.$$

The manual annotation of a noise-free and noisy segment of the complete signal is distinguished based on the feature points of the ECG signal. Any ECG segment with clear presence of all the feature points is labelled as noise-free segment. By contrast, the absence of any feature point in a segment due to the presence of noise is labelled as a noisy segment.

## 4. Results

### 4.1. Effect of dataset diversity on statistical signal quality indices

**Figure 4.**
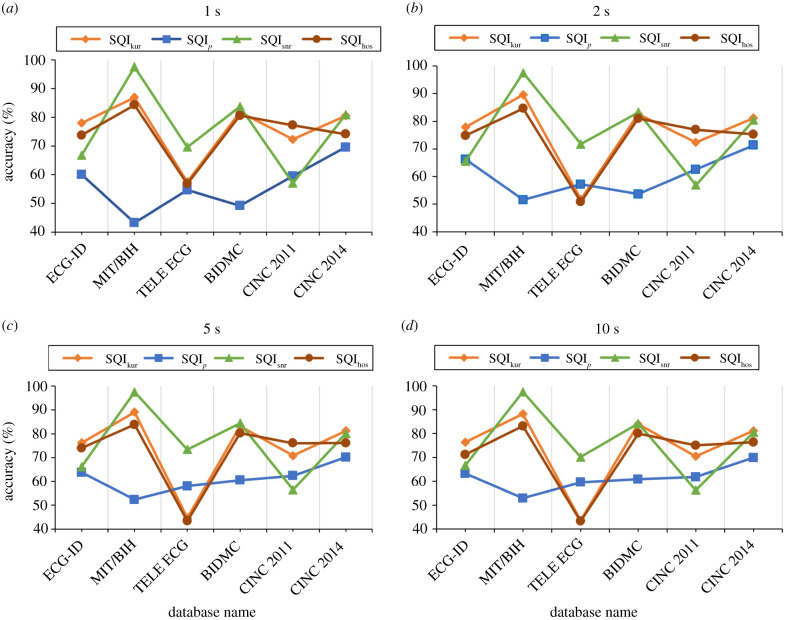
The impact of dataset diversity on SSQIs accuracy rate in four window sizes. The SQI_kur_ and SQI_hos_ are constant across the datasets compared to SQI_*p*_ and SQI_snr_: (*a*) 1 s segment SSQI is close to 80% except Tele ECG, CINC 2011 and 2014, (*b*) 2 s SQI_Snr_ performance increasing for Tele ECG, (*c*) 5 s MIT/BIH shows the highest accuracy rate for SQI_snr_ and (*d*) 10 s segment Tele ECG accuracy rate decrease for SQI_snr_.

The impact of dataset diversity on the accuracy of the four studied SSQIs is demonstrated in figure 4 and summarized in table 5.

**Table 5.** Impact of dataset diversity on four statistical SQIs (SSQIs) such as kurtosis (SQI_kur_), relative power ratio of QRS complex (SQI_*p*_), high-order statistic (SQI_hos_) and signal-to-noise ratio (SQI_snr_) based on accuracy (Acc), specificity (*S*_*p*_) and sensitivity (*S*_*e*_). Bold value indicates the best performance for each SSQIs in different datasets.

dataset	window size (s)	SQI											
		SQI_kur_			*SQI*_*p*_			SQI_snr_			SQI_hos_		
		*Se* (%)	*Sp* (%)	Acc (%)	*Se* (%)	*Sp* (%)	Acc (%)	*Se* (%)	*Sp* (%)	Acc (%)	*Se* (%)	*Sp* (%)	Acc (%)
ECG-ID	1	93.34	45.54	**77.96**	75.99	26.4	60.03	94.48	8.23	**66.73**	90.66	37.98	73.71
	2	93.48	44.96	77.87	91.14	13.78	**66.25**	94.08	5.14	65.47	91.22	40.2	**74.81**
	5	93.09	40.69	76.23	89.88	8.88	63.82	95.09	5.18	66.16	91.45	37.23	74.01
	10	95.23	36.56	76.35	89.67	7.57	63.25	95.9	5.18	66.71	89.81	32.05	71.23
Tele ECG	1	38.96	**89.95**	57.48	50.79	61.36	54.63	**99.74**	16.72	69.58	38.10	89.60	56.81
	2	30.23	89.10	51.74	56.22	58.73	57.14	99.70	23.11	71.72	28.73	89.15	50.80
	5	19.89	86.61	44.65	57.41	59.16	58.05	98.22	31.32	**73.40**	18.33	85.96	43.43
	10	22.76	75.40	43.48	56.89	63.74	59.58	95.68	30.88	70.17	19.86	79.29	43.26
BIDMC	1	84.13	27.83	81.81	50.27	23.92	49.18	87.07	5.29	83.70	82.96	25.53	80.59
	2	84.97	28.14	82.63	55.09	18.84	53.59	86.52	6.89	83.24	83.27	28.37	**81.00**
	5	85.68	26.6	83.24	61.83	30.39	60.54	87.76	6.71	**84.43**	82.46	29.65	80.29
	10	86.51	24.24	**83.95**	62.3	26.99	**60.85**	87.57	6.18	84.22	82.31	26.64	80.02
MIT/BIH arrhythmia	1	88.05	41.62	86.89	42.77	60.91	43.23	99.99	0.63	97.50	85.24	46.47	84.27
	2	90.88	38.82	**89.58**	51.49	54.96	51.57	99.99	0.84	**97.51**	85.63	46.3	**84.64**
	5	90.33	42.03	89.12	52.33	52.82	52.34	99.97	1.29	97.50	84.67	51.47	83.84
	10	89.47	41.09	88.26	52.88	54.05	**52.91**	99.94	0.39	97.45	84.02	50.26	83.17
CINC 2011	1	87.5	30.22	72.29	66.42	40.28	59.48	74.77	7.89	**57.01**	93.86	31.11	**77.2**
	2	88.47	27.69	**72.33**	73.23	33.01	**62.55**	74.54	8.10	56.89	94.35	28.86	76.96
	5	88.76	21.17	70.81	73.56	31.53	62.40	74.59	6.50	56.50	94.59	24.9	76.08
	10	90.05	16.37	70.49	73.32	29.96	61.80	73.92	7.62	56.31	95	19.97	75.07
CINC 2014	1	98.3	2.92	80.51	82.18	14.36	69.52	98.88	2.50	**80.89**	90.16	4.29	74.13
	2	99.26	2.24	81.16	86.26	6.35	**71.35**	98.74	0.29	80.37	91.73	3.65	75.29
	5	99.55	1.38	**81.23**	84.78	6.33	70.14	98.55	0	80.16	92.91	3.01	76.13
	10	99.62	0.54	81.12	84.56	6.04	69.90	**98.85**	0.01	80.40	93.31	2.62	**76.38**

From table 4 and table 5, it can be observed that SQI_snr_ achieves the maximum accuracy of 97.51% when using MIT/BIH arrythmia datasets. In addition, we can see that SQI_kur_ preforms reasonably well across all the datasets and obtains more than 72% accuracy. In table 5, window sizes are adjusted to obtain maximum accuracy for each SQI and datasets.

From figure 4, we can quantify the maximum and minimum best accuracy of SQI_kur_, SQI_*p*_, SQI_snr_ and SQI_hos_ across all datasets from these values and they are (89.58%, 72.33%), (71.35%, 52.91%), (97.51%, 57.01%) and (84.64%, 74.81%). From these ranges and variations of accuracy across different datasets (as shown in figure 4), it is obvious that the SQI_kur_ and SQI_hos_ are more consistent than other SQIs for accurately differentiating noisy ECG segment from the clean ones.

The highest accuracy for individual datasets ECG-ID, BIDMC, MIT/BIH arrythmia, Tele ECG, CINC 2011 and CINC 2014 was obtained using SQI${\;}_{\mathrm{kur}}(77.96\text{\%})$, SQI${\;}_{\mathrm{snr}}(84.43\text{\%})$, SQI${\;}_{\mathrm{snr}}(97.51\text{\%})$, SQI${\;}_{\mathrm{snr}}(73.40\text{\%})$, SQI${\;}_{\mathrm{hos}}(77.20\text{\%})$ and SQI${\;}_{\mathrm{kur}}(81.23\text{\%})$ for window size 1 s, 2 s, 5 s, 5 s and 10 s, respectively.

On the other hand, the specificity (*S*_*p*_), which is the key parameter for measuring the misclassification rate of noisy signals, of SSQIs across the datasets is not promising as expected in clinical usability (shown in table 5). The maximum specificity (*S*_*p*_) of SQI_kur_, SQI_*p*_, SQI_snr_ and SQI_hos_ for six datasets are (45.54%, 28.14%, 42.03%, 30.22%, 2.92%), (26.40%, 30.39%, 60.91%, 40.28%, 14.36%), (5.18%, 6.89%, 1.29%, 8.10%, 0%) and (40.20%, 29.65%, 51.47%, 31.11%, 4.29%). This indicates that the SSQIs-based signal quality assessment approach is biased towards detection of noise-free signals.

### 4.2. Effect of window size on statistical signal quality indices

**Figure 5.**
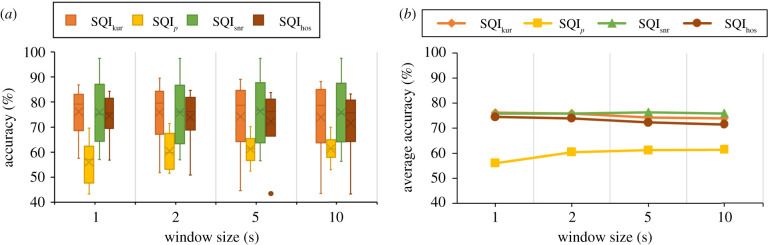
Impact of window size on four statistical SQI (SSQIs): (*a*) IQR-mean boxplot represents the average accuracy rate of six datasets for four SSQIs. Kurtosis (SQI_kur_) and signal to noise ratio (SQI_snr_) shows the consistency in four window sizes. However, relative power ratio (SQI_*p*_) and high-order statistical SQI (SQI_hos_) fluctuated over the window sizes. The dot point in the figure means the outlier of the boxplot. Outliers are the data points that differ from other datasets’ accuracy rates. (*b*) Line graph delineates average accuracy rate across the window size where SQI_snr_, SQI_kur_, SQI_hos_ are close to each other. However, SQI_*p*_ shows the lowest accuracy rate among all the SSQIs.

The impact of diverse window size on the accuracy rate for four SSQIs is shown in figure 5 and table 6. The SQI_kur_ achieved the highest accuracy for 1 s and 2 s (76.16%, 75.88%), and SQI_snr_ achieved the highest accuracy for 5 s and 10 s (76.36%, 75.88%) window. By contrast, SQI_*p*_ showed the lowest accuracy across all the window sizes. SQI_hos_ showed second and third best accuracy and their performances are consistent across all the window sizes that can be seen from figure 5 and table 6.

**Table 6.** Impact of window size diversity on four statistical SQI (SSQI) such as kurtosis (SQI_kur_), relative power ratio of QRS complex (SQI_*p*_), high-order statistic (SQI_hos_) and signal-to-noise ratio (SQI_snr_) based on accuracy (Acc), specificity (*S*_*p*_) and sensitivity (*S*_*e*_). Bold value indicates the best performance for each SSQIs in different datasets.

window size (s)	SQI											
	SQI_kur_			SQI_*p*_			SQI_snr_			SQI_hos_		
	*Se* (%)	*Sp* (%)	Acc (%)	*Se* (%)	*Sp* (%)	Acc (%)	*Se* (%)	*Sp* (%)	Acc (%)	*Se* (%)	*Sp* (%)	Acc (%)
1	81.71	39.68	**76.16**	61.40	37.87	56.01	92.49	6.87	75.90	80.16	39.16	74.45
2	81.21	38.49	**75.88**	68.91	30.94	60.41	92.26	7.40	75.87	79.15	39.42	73.92
5	79.55	36.41	74.21	69.96	31.52	61.21	92.36	8.50	**76.36**	77.40	38.70	72.30
10	80.61	32.37	73.94	69.94	31.39	61.38	91.98	8.38	**75.88**	77.39	35.14	71.52

The accuracy of SQI_snr_, SQI_kur_ and SQI_hos_ are close to each other as depicted in figure 5*b*. Therefore, it is difficult to interpret the best SSQIs for different window sizes. The IQR-mean boxplot, which is shown in figure 5*a*, is one of the ways to select the best SSQI by assessing the variability for mean, maximum and minimum accuracy in the boxplot. Among all the SSQIs, SQI_snr_ is better due to the maximum accuracy and prominent mean value compared to SQI_kur_ and SQI_hos_. Figure 5*b* shows that the SQI_snr_ holds the highest average accuracy. In terms of individual accuracy, SQI_snr_ is also higher than SQI_kur_. As a result, the SQI_snr_ is optimum among all the SSQIs. However, SQI_*p*_ shows the lowest accuracy (61.38%) among all the SSQIs. The SQI_kur_ achieved the highest average accuracy, which is 75.16% for the 1 s window. In the case of specificity (*S*_*p*_), the SQI_kur_ is better than other SSQIs at 39.68%. However, this result is not promising due to poor detection of TN and high FP detection. In a clinical case, FP is to be avoided. Hence, it requires further analysis of SSQIs to increase the specificity (*S*_*p*_), which is important for clinical analysis.

## 5. Discussion

The adoption of wearable sensors in clinical settings are limited mostly due to the lack of reliability of those sensors in capturing the signal without artefacts. Although most of the wearable sensors continuously record the signal, they are unable to label the noisy or noise-free section that can be used by decision-making systems. One way to add this feature in the wearable devices is building automatic methods for detecting noise in the signal. Most of the previous studies, investigated SSQIs for a single window size (mostly 10 s) and validated using a limited sets of labelled segments [48,58–60]. As a result, the applicability of these indices, especially the use of single threshold, across a wide range of ECG datsets is yet to be explored. Therefore, in this study, we have comprehensively investigated the performance and limitations of these well-defined statistical approaches for noise detection in ECG signals. We empirically evaluated existing SSQIs with respect to varying segment length and diverse datasets. The major findings of this study are

(i) Most of the SSQI algorithms show very high accuracy across varying window sizes; however, their specificity values are very low;
(ii) The performance of SSQIs are more difficult to generalize over datasets than the window size.

### 5.1. Effect of dataset diversity on statistical signal quality indices

The effect of dataset diversity on SSQIs is one of the major concerns for selecting the optimal one for any given application. Performance analysis on large-scale experimental datasets is the proper way to generalize the capacity of SSQIs. Data collected through different experimental set-ups better represents the dataset diversity, which primarily includes one or more of the following: (a) number of channels; (b) location of electrodes; and (c) sampling frequency. Therefore, selection of a large number of datasets is better than using a longer recording length from a single dataset to select the best performing SQI.

Table 2 outlines the dataset and dataset size used by this study and popular related studies in the literature. From table 2, it is clear that this study uses the largest range of datasets for analysing performance of SSQIs compared to previous studies. Therefore, we believe that the outcome of this study is more reliable and dependable than the existing studies.

In this study, we generalized the impact of dataset diversity on four SSQIs: SQI_kur_, SQI_*p*_, SQI_snr_ and SQI_hos_. From figure 4 see that SQI_*p*_ has the lowest accuracy across all the datasets. One of the reasons behind this lowest performance is the dependency of SQI_*p*_ measure on the frequency range (0–15 Hz). For capturing power of the very low-frequency components (<1 Hz), a longer window size is usually required. Therefore, in contrast to other SSQIs, SQI_*p*_ is biased towards capturing noise and as a result, the sensitivity is low. Since the dataset is biased (skewed towards noise-free segments), this has directly affected the accuracy of SQI_*p*_. Based on these results, we can conclude that SQI_*p*_ is mostly unsuitable for noise detection using short-length windows. SQI_*p*_ may not perform well for pathological ECG signals, since their frequency responses are different from normal and may affect the power distribution in 0–15 Hz.

SQI_kur_ and SQI_hos_ exhibit better generalization capability across all the datasets. However, SQI_kur_ and SQI_hos_ give very high index values in the presence of high-frequency noise. As a result, ECG signals with high-frequency noise are classified as clean signals, which decrease the specificity of these SSQIs (table 5). The specificity needs to be improved for clinical decision-making and therefore, further research is necessary to overcome this issue.

The most surprising result is shown by SQI_snr_, where the performance varies significantly with varying datasets. SQI_snr_ has shown the highest accuracy among all studied SSQIs for MIT/BIH arrhythmia; however, accuracy drops to a very low value for the Tele ECG dataset. One of the reasons is that the measurement bias of SQI_snr_ towards noise-free signals. The ratio of noise-free and noisy epochs is very high for MIT/BIH arrythmia and very low for Tele ECG as shown in table 3. We have also observed that SQI_snr_ is very vulnerable to noise. As a result, it showed relatively better performance for ECG-ID though the ratio of noise-free and noisy epoch is very low.

From the observed performances of SSQIs on different datasets, we can conclude that there is no single SQI index that works or shows generalization capacity across varying datasets. In addition, despite showing high accuracy, all these indices resulted in very low specificity, which indicates that they are not suitable for embedding in clinical decision-making systems. The inherent limitation of these methods can be defined as the use of static threshold value for filtering noise-free signal from the noisy signal. Therefore, further investigation is necessary to address the limitations such as measurement bias and static threshold.

### 5.2. Effect of window size on statistical signal quality indices

As observed in this study, the performance of SSQIs is less dependent on the window size than the variation of dataset as shown in figure 5*a*,*b*. SQI_*p*_ is found to be more sensitive towards the window size, however that is only visually distinguishable for one second window size. As described in §5.1, the dependency on the frequency range may affect the performance of SQI_*p*_ for the small size window (figure 5*b*). SQI_kur_, SQI_hos_ and SQI_snr_ have a constant accuracy rate across all window sizes as illustrated in figure 5*b*. However, the specificity of all SSQIs decreases consistently with increasing window size. One reason for this can be partial presence of noise in the whole segment of signal rather than a completely noisy signal. Therefore, it is better to use a small window size rather than a larger one. The high variability of SQI_snr_ and SQI_*p*_ represents the effect of dataset rather than window size.

This study explains the limitation of existing threshold-based SSQIs for diverse datasets and window sizes. The reasons for selecting these SSQIs are low computational cost, which makes them energy-saving indices and easy to deploy in resource-constrained devices. However, it is essential to generalize the SSQIs threshold values so that they can be applied to diverse datasets. Our finding suggested that an adaptive threshold or machine-learning model should be used to eliminate this threshold limitation. However, while developing an alternative solution, computational cost and deployability in the resource-constrained device should be considered.

## 6. Future directions

In this section, we provide details of future directions related to SQIs.

### 6.1. Improving the performance of classification mechanisms

#### 6.1.1. Generalized threshold

Instead of using small ECG datasets for determining a threshold value, larger datasets should be used to find generalized threshold values that should able to separate noise and noise-free ECG segments.

#### 6.1.2. Classical machine-learning approaches

Classical machine-learning approaches can be used for learning the nature of noise in ECG signals and automatically recognize noisy segments from the training model. Machine-learning models depend on the signal feature instead of amplitude threshold value. Therefore, in the machine-learning model, we can eradicate the noise separation threshold in the ECG signals.

#### 6.1.3. Deep learning model

Another possible solution for identify noise in ECG signals is to use deep learning models where noisy and noise-free segments are learnt from raw segments instead of signal features. Therefore, there is no need to rely on features such as linear and nonlinear features.

#### 6.1.4. Developing adaptive threshold values

The performance of SSQIs is clearly dependent on the selection of thresholds values. From our simulations, we found that one fixed threshold value is not ideally suited to all the ECG datasets. Therefore, ECG signal feature-dependent threshold values should be assigned dynamically.

### 6.2. Development of application specific electrocardiogram signal classification models

#### 6.2.1. Deploy model in the real-time device

Off-line and on-line model testing are important to verify model performance for noisy and noise-free ECG segment detection. Real-time noise detection in ECG signals is a very challenging task.

#### 6.2.2. Development of flexible noise detection algorithms

There is a trade-off between computational complexity and accuracy of noise detection algorithms used. Flexible, noise detection algorithms should be developed that depend on the requirements of the applications, it should be possible to achieve higher computational efficiency or accuracy.

### 6.3. Development of noise adaptive electrocardiogram signal classification algorithms

#### 6.3.1. Formally defining noisy electrocardiogram signals

As mentioned previously, ECG signals contain vital information related to the condition of the heart. Therefore, with the help of healthcare professionals, a formal definition of ECG noise should be developed. This will assist in developing efficient and automated mechanisms to detect noise in the ECG signals.

#### 6.3.2. Finding the relationship between electrocardiogram signals and performance of statistical signal quality indices

From our work, we found out that performance of SSQIs varies significantly across different datasets. Future work is necessary to find out the relationship between the ECG signals features (such as frequency content) and SSQIs.

## 7. Conclusion

Automated signal quality assessment is one of the key components for developing wearable or remote monitoring solutions. Wearable devices are very prone to noise, and it is important to detect the noisy signal appropriately, so that unnecessary transmissions and further processing are reduced. Another importance of SSQIs is to ensure the quality of the signal before use by a decision-making system, since accuracy of such a system depends on signal quality. This study explores the robustness of commonly used SSQIs. To evaluate the performance of SSQIs, we annotated the dataset using knowledge from existing studies by considering their pathological features such as P, Q, R, S and T peak values, which is mentioned in the dataset labelling section. We preformed extensive simulations to asses the robustness of SSQIs for varying window sizes across different datasets. We have found that, while the performance is highly sensitive to different datasets, the window sizes have minimal affect on performance. We strongly believe that these suggestions will assist current and future researchers. Although this study highlights only the robustness of automated signal quality assessment methods, computational cost and energy consumption are also key parameters for developing an efficient method for wearable devices. Thus, those aspects should be considered while proposing an alternative method for signal quality assessment in wearable devices.

## Data Availability

Data are available at https://github.com/Innovation-in-Healthcare/Robustness-of-Electrocardiogram-ECG-S.
